# Oxidative Stress in Peripheral Arterial Disease (PAD) Mechanism and Biomarkers

**DOI:** 10.3390/antiox8090367

**Published:** 2019-09-02

**Authors:** Salvatore Santo Signorelli, Salvatore Scuto, Elisa Marino, Anastasia Xourafa, Agostino Gaudio

**Affiliations:** Department of Clinical and Experimental Medicine, University of Catania, 95125 Catania, Italy

**Keywords:** peripheral arterial disease, pathophysiology, oxidative stress, biomarkers, inflammation

## Abstract

Hemodynamic dysfunction mainly characterizes pathophysiology of peripheral arterial disease (PAD) leading to chronic ischemia. Hemodynamic dysfunction is the origin of intermittent claudication (chronic PAD) or of critical limb ischemia (very severe PAD). Notably, it is well known that oxidative stress (OxS) plays a pathophysiological role in PAD. The higher production of reactive oxygen species (ROS) from OxS and reduced redox capability are two crucial players in initiating and progressing PAD. A number of biomarkers highlight OxS and monitor it in PAD. The present review summarizes data on OxS, on biomarkers available to mark OxS occurrence and to monitor on PAD progression, as well as to evaluate the effects treatments in PAD patients. In conclusion, by detailing OxS and its biomarkers, we hope to encourage more studies to focus on drugs which combat OxS and inflammation.

## 1. Introduction

Peripheral arterial disease (PAD) is one clinical aspect of atherosclerotic disease, presenting impaired blood flow in the lower limbs and where intermittent claudication is the most significant symptom (Table 1) [1,2]. PAD patients have a high risk of cardiovascular disease (CVD) morbidity [3,4], because they suffer from several arterial co-morbidities (i.e., coronary artery disease, carotid ischemic diseases causing a risk of ischemic stroke) [5,6]. Atherosclerosis can begin in young adults with the development of fatty streaks [5]. Smoking and type 2 diabetes mellitus represent the two major risk factors predisposing to PAD [1,2,3,4,5,6,7,8]. Moreover, dyslipidemia, hypertension, endothelial dysfunction [9], and pro-inflammatory molecules [10,11] contribute to atherosclerotic diseases. Epidemiology of PAD ranges to more than 200 million of subject worldwide, it affects a mainly affects 60–65 year-old individuals thus affecting more than 20 million patients in Europe and North America [12,13,14,15,16]. PAD mainly affects 60–65 year-old individuals thus affecting more than 20 million patients in Europe and North America. Atherosclerosis is an inflammatory disease so inflammation has a crucial role in promoting, initiating and progressing PAD [13,14,15,16,17]. A number of inflammatory biomarkers (i.e., acute phase proteins, C reactive protein, fibrinogen, pro-inflammatory cytokines) have been found to be associated with PAD. Moreover, oxidative stress (OxS) seems to play a pathophysiological role in PAD [18,19,20]. OxS leads to the accumulation of post-translational bio-molecules (e.g., protein carbonylation or aldehyde/ketone adducts, nitration and sulfoxidation, DNA lesions such as 8-oxodG), furthermore interfering with physiological redox capability. Reactive oxygen species (ROS) and nitrogen species (RNS) multiply in many chronic diseases whereas redox regulation lowers them. There is a close association between OxS and cardiovascular diseases, oxidative stress biomarkers marking their progression.

### 1.1. Oxidative Stress and PAD

**Figure 1:**
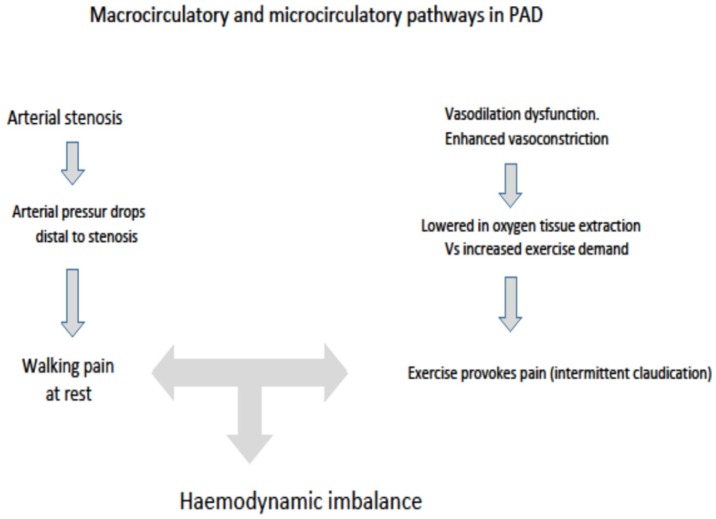
Macrocirculatory and microcirculatory mechanisms in compromising walking capability in peripheral chronic ischemia.

**Figure 2:**
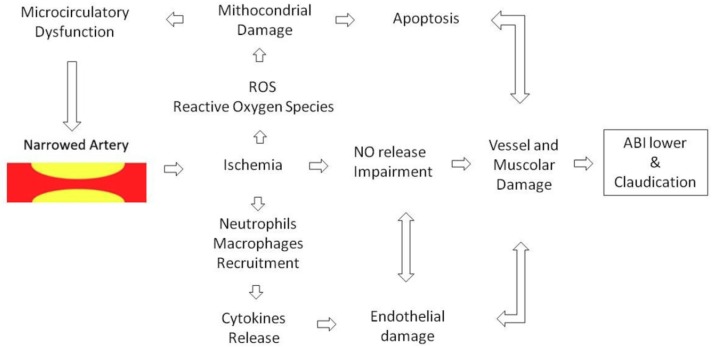
Pathophysiology of peripheral arterial disease.

The close relationship between OxS and the atherosclerotic process have been widely investigated [21,22,23,24]. On OxS in PAD, it has been proposed since 1977 that oxidation processes may play a role in PAD pathophysiology [25]. A number of surrogate (indirect) oxidative markers were sought for PAD. Results of studies highlighted on several markers of oxidative stress such as beta 2 microglobulin [26], high sensitivity C-reactive protein [27] and cystatin C [28]. Most recently, other biochemical indices as the chemochil ligand 2, subclasses of isoprostanes [29] were proposed as useful in responding about role played by oxidative stress and inflammation in PAD pathophysiology and in diagnosing more favorable biochemical pathway for PAD. On OxS as a pathophysiological key in PAD, it enhances the inflammatory pathways responsible for the atherosclerotic process [30,31,32,33,34,35,36], which may be summarized as the downstream outcome of the inflammatory cytokines which promote lipid and protein oxidation in artery walls. Low-density lipoprotein (LDL) oxidation is the initial step for the atherosclerotic process. OxS is closely related to impaired nitric oxide (NO) synthesis [31]. The lack of vasodilation in the narrowed atherosclerotic arteries of the lower limbs [37,38] is the precursor to ischemic injury to tissues (Figure 1) and cells leading to the multiplication in the reactive oxygen species (ROS) caused by the dysfunction of NO bio-availability [39,40]. The bio-availability of ROS is known to result from the rate of ROS formation, above all by the mitochondria, and from its rate of clearance through the antioxidant defense system [40]. ROS cooperates with OxS leading to the progressive dysfunction of the antioxidant defense system as shown in studies where there is a significant decrease in the activity of the principal enzymatic antioxidants [40,41,42,43,44]. Additionally, OxS promotes endothelial dysfunction, which in turn decreases NO production impairing its protective role. This initiates a dangerous mechanism [45] which leads to accelerating atherosclerosis (Figure 2). Reperfusion injury is a crucial issue in the chronic ischemia of PAD. PAD patients experience a severe reduction in blood flow during muscular activity, followed by reperfusion when the muscular stress ceases. Reperfusion increases the ROS production by mitochondria, and the increased OxS in turn causes injury to ischemic muscles [45,46]. PAD patients suffer from the myopathy characterized by a decrease in myofibre cross-sectional area [47]. Indeed, metabolic myopathy and OxS plays a crucial role in the pathophysiology of PAD operating as mechanisms behind the structural and metabolic changes occurring in ischemic muscles. [48] To learn more about OxS, several biomarkers have been proposed as helpful tools in evaluating more prone individuals and in monitoring PAD outcomes. These OxS biomarkers have proven to be closely correlated to the progression and severity of arterial disease [39,47,48,49]. A number of clinical trials have been carried out to evaluate the therapeutic effects of anti-oxidant therapies (glutathione, vitamins E and C, carnitines) against OxS damage in PAD [48,49,50,51].

### 1.2. Surrogate Biomarkers in PAD

OxS has been closely associated with cardiovascular diseases, oxidative biomarkers correlating with disease progression. OxS biomarkers have proven helpful in improving the early diagnosis of PAD, particularly in asymptomatic patients [52]. Currently, imaging techniques (US, CT and MRI) are the preferred diagnostic instrument for PAD and its monitoring [53,54,55]. The ankle–brachial index (ABI) measurement is widely agreed as an easy and repeatable method for PAD diagnosis as well as management [53]. Having completed the research on reliable biomarkers, it may be interesting to identify asymptomatic PAD patients or those at higher-risk to seek for PAD. Although the role of OxS biomarkers in clinical practice has been evaluated, it is still under discussion. We have summarized the findings on surrogate OxS biomarkers in PAD in the following sections.

#### 1.2.1. Malondyaldheide (MDA)

Antioxidants are classified into two groups according to the mechanism by which they prevent or retard oxidation. The primary (chain-breaking) antioxidant group (e.g., c*-tocopherols) acts by interrupting oxidation and converting free radicals to stable species. The secondary antioxidant (e.g., ascorbic acids) group reacts with oxygen before the start of oxidation. The oxidation rate may be evaluated by generating hydroperoxides (per minute and per milligram of low density lipoprotein). Another surrogate biomarker of OxS is malondialdehyde concentration. MDA plasma levels differ between PAD and healthy controls as well as MDA levels being higher in PADs than in non-PAD individual before starting a treadmill test [56,57]. Maximal muscle stress enhances differences in MDA levels. However, anti-oxidant drug treatment (by propionil-L-carnitine i.v.) initiates reductions in the plasma levels of these oxidative makers both at rest and after treadmill tests. There is reasonable agreement on the helpful role of oxidative biomarkers in highlighting OxS in PAD, so OxS must be targeted in treating PAD patients [58,59,60].

#### 1.2.2. 4-Hydroxynonenale (4-HNE)

4-HNE (4-Hydroxy-2-nonenal) is generated by the peroxidation of n-6 polyunsaturated fatty acid. HNE has a number of biological properties mainly represented by HNE-adduct generation on free amino groups and thiol groups on proteins. HNE adduct accumulation initiates cellular dysfunction, such tissue damage being a huge factor in atherosclerosis and arterial atherosclerotic diseases. HNE contributes to the atherogenicity of oxidized LDL by deviating the LDL metabolism and leading it to generate foam cells through the scavenger receptor pathway of macrophage cells. HNE de-regulates cellular responses at higher concentrations causing inflammatory responses and apoptosis. HNE is involved in re-modelling atherosclerotic plaque through the progressive modification of smooth muscle cells, also effecting proliferation, angiogenesis, and cell apoptosis. HNE adducts in the core of atherosclerotic plaque contribute to macrophage and smooth muscle cell apoptosis which in turn lead to a high risk of athero–thrombotic events. There is data on the accumulation of biogenic toxic aldehydes in critical ischemic tissues such as in ischemia-reperfusion injury. Notably, 4-hydroxy-2-nonenal (4-HNE) is a highly reactive aldehyde with abundant adduct formation which negatively effects contractility and the mitochondrial energy of muscle cells. High levels of 4-HNE modified proteins were found in PAD patient muscles such that the accumulation of the derived aldehyde product (4-HNE) is now considered as a marker for skeletal muscle injury initiated by OxS [61,62].

#### 1.2.3. Micro RNAs (mRNAs)

While the peculiar role played by endothelial cells on correct function of circulation is clearly known, on the other hand, it is known that vascular smooth cells communicate with endothelial cells via mRNAs. Such mRNAs (i.e.,143 and 145) promote cell-to-cell interplay, finally favoring specific effects. Transport of delivered mRNAs to endothelial cells has been shown by using a high resolution imaging method [63].

mRNAs 143/145 play a key role for maintaining contractile phenotype of vascular smooth cells (VSMC) dysregulation of vascular tone and of arterial pressure were demonstrated in mRNAs-143/145−/−deficient mice. It is clear as the mRNAs show different capabilities both in contrasting or in maintaining functions of endothelial cells leading to equilibrium of vascular tone [64].

Capability of endothelial cells on circulatory equilibrium must achieve aiming against haemodynamic dysfunction and its unbalance is directly favorable to provoking arterial damage. Such mRNAs as mRNA-126 released by the endothelial is internalized by white cells (monocytes) and vascular smooth cells leading them to play a role to against endothelial dysfunction. There is a clear explanation of the crucial protective capability of mRNAs 126 from study on diabetes having highly prevalence of arterial consequences also linked to endothelial cell dysfunction [65,66,67,68]. Cell-to-cell talk between VSMC with endothelial cells promotes activation of mRNAs, its transcription thus mRNAs are transferred to endothelial cells. In patients with ischemic coronary diseases there is a raised circulating level of mRNA 126 but it is noteworthy to prevent the deleterious effects of endothelial cells [69]. mRNAs are emerging biomarkers for assessing oxidative stress. We have shown that miRNA values significantly increase in patients with PAD compared to controls [70]. These results confirm those of previous studies which show increased MiRNAs values in patients with progressive atherosclerosis compared to controls [71]. mRNAs may play a key role in the adaptive mechanism of maintaining cellular homeostasis during ischemic injury [68,69]. Their production is stimulated by Nrf2, a fundamental nucleic factor involved in intracellular redox balance [71,72].

#### 1.2.4. Isoprostanes

Markers for the oxidation of LDL and eicosanoid formation (e.g., isoprostanes) were among the first to be analyzed. Isoprostanes derive from the metabolism of arachidonic acid induced by free-radical catalyzed processes [73]. Several previous studies have proven the reliability of 8-iso-prostaglandin F_2α_ (a subtype of F2-isoprostanes, the most plentiful subclass) as a dependable method for evaluating oxidative stress in PAD patients [73,74]. The plasma values of F2-isoprostanes were higher in PAD patients than in controls. Our group reported a contradictory result on F2-isoprostane values in PAD patients which were lower compared to controls [74]. This outcome is associated with a significant increase in micro-RNAs suggesting an adaptive response to reduce oxidative stress.

#### 1.2.5. Paraoxonase

Among the most significant biomarkers of oxidative stress with the role of antioxidants is Paraoxonase (PON): this family of enzymes protects lipoprotein from peroxidation [72]. 

Particularly in PAD patients compared to no PAD individuals were demonstrated lower paroxonase-1 (PON-1) concentration whereas PON-1 activity was raised. As we know, PON-1 activity is strongly dependent on the oxidation of lipoproteins, and in PAD patients there is a compensatory mechanism in favoring protective of PON family to against peroxidation [73]. PON is most abundant in the circulation, and may be considered as a novel marker of arteries status, and as a helpful marker of oxidative stress in PAD with multiple atherosclerotic diseases and in PAD patients [75,76,77,78].

#### 1.2.6. Nrf2/Heme-oxygenase 1 (HO-1)

Transcriptional factor Nrf2 is one of the most important regulator molecules in the antioxidant system, appearing to protect against reperfusion injury. It promotes the transcriptional activation of several antioxidant (ARE) binding responses [30]. Nrf2 is also a fundamental regulator of heme oxygenase-1 (HO-1), an enzyme that promotes protection against oxidative stress [79] and also contributes to angiogenesis. It catalyzes the breakdown of heme to CO, ferrous iron and biliverdin [79]. Biliverdin is reduced to bilirubin which protects against the oxidation of lipids. The exact role of HO-1 in PADs remains to be investigated. It seems to protect endothelial cells by the effect of hypertension, oxidative stress and inflammation [80]. In particular, the increased production of CO intensifies its effects of vasodilatation and anti-inflammatory activity [81]. Values of HO-1 are lower in PAD patients, and it has been suggested as an independent predictor of PAD [81]. It is likely that lower HO-1 is part of a compensatory mechanism to maintain cellular redox balance, and HO-1 may be a reliable marker in the diagnosis and prognosis of PAD. Among the biochemical responses to OxS, the heme oxygenase (HO) system has been suggested as key [82]. It is known that HO-1 is an intracellular enzyme that catalyzes the breakdown of heme to carbon monoxide, ferrous iron and biliverdin [82,83,84]. Numerous effects of potential significance to the cardiovascular system have been reported, including protection against ischemia/reperfusion [79], blood pressure regulation [23,85,86,87], inflammation [80,81] and in angiogenesis [83]. HO-1 may be released into the plasma of smooth muscle cells, cardiomyocytes, leukocytes, monocytes/macrophages and/or endothelial cells that are damaged by the effect of hypertension, oxidative stress and/or chronic inflammation [23,82,83,84,85,86,87]. We demonstrated low HO-1 plasma in PAD patients but no relationship was found between HO-1 plasma levels with the progressive stages of PAD [79]. Interestingly, in regression analysis we found a close relationship between low levels of glutathione as a redox marker for the severity of PAD. Reduced HO-1 plasma levels demonstrated an impairment in the protective properties of HO-1 in PAD patients [74,79].

## 2. Conclusions

It is known that OxS plays a role in atherosclerotic processes as well as in PAD, thus it is reasonable that OxS must be targeted by using multiple approaches. High levels of OxS biomarkers were found in PAD patients with chronic ischemia causing intermittent claudication and in critical limb ischemia patients showing more severe ischemia of the peripheral arteries. OxS and pro-inflammatory conditions increased in PADs both of which are crucial in determining endothelial dysfunction. It is widely accepted that all the aforementioned conditions accelerate the atherosclerotic process. To date, endothelial dysfunction is considered an independent risk factor for morbidity and mortality in PAD patients. It is worth remembering that PAD is a predictive clinical marker for extended atherosclerotic processes in other arteries. From a therapeutic point of view, physical and pharmacological options are mandatory to reduce or counteract OxS and inflammation. On the other hand, to date we note the positive effect originated by no pharmacological strategy for PAD as the supervised physical training. It reduces OxS as well as the plasma levels of inflammatory biomarkers in parallel with improved walking distances measured by the treadmill test. These findings are very intriguing because improved muscle and physical performance may be crucial issues for PAD patients. It is clearly accepted that a well-structured rehabilitation program, may improve both performance, quality of life furthermore walking capability effects on the functional balance of patients with PAD [88,89,90,91,92]. However authors highly endorse the evaluation of the efficacy of muscle exercise in threatening PAD, suggesting awareness of potential damage to calf muscle fibers derived by strenuous physical exercise during treadmill training. A number of drugs have demonstrated their anti-oxidant capability in PAD patients in results from studies on their clinical efficacy in PAD. However, we still want to call for action more studies focusing on anti-oxidant and anti-inflammatory agents to achieve the aforementioned targets as well as to ameliorate walking distance, lower cardiovascular morbidity and mortality in PAD patients. A combined approach including anti-oxidant drugs, physical training, and re-vascularization may be effective against the multiple pathological modifications induced by PAD [92,93].

## Figures and Tables

**Table 1:** Progression and staging of peripheral arterial disease (PAD) by based on two most applied classifications.

Fontaine’s Classification [1].			Rutherford’s Classification [2].		
Stage	Clinical	Symptoms	Pathophysiology	Clinical	Grade
1st	no symptoms	occasional discovery of aortic and iliac calcifications	ats plaque risk plaque inflammation	asymptomatic	0/0
2nd A	claudication	absolute claudication distance > 200 mt recovery t. < 2 min	discrepancy oxygen request arterial supply	mild claudication moderate claudication	I/1 I/2
2nd B	claudication	ACD < 200 m recovery time > 2 min ACD < 100 m recovery time > 2 min	discrepancy oxygen request arterial supply Highest discrepancy and acidosis	severe claudication	I/3
3rd	ischaemic rest pain	ischaemic rest pain	skin hypoxia acidosis	ischaemic rest pain	II/4
4th	ulceration or gangrene	Skinn necrosis gangrene	severe skin hypoxia acidosis	minor tissue loss major tissue loss	III/5 III/6
